# Influence of the photodeposition sequence on the photocatalytic activity of plasmonic Ag–Au/TiO_2_ nanocomposites†

**DOI:** 10.1039/d2na00440b

**Published:** 2022-08-31

**Authors:** Bela D. Bhuskute, Harri Ali-Löytty, Mari Honkanen, Turkka Salminen, Mika Valden

**Affiliations:** Surface Science Group, Faculty of Engineering and Natural Sciences, Tampere University P.O. Box 692 FI-33014 Tampere Finland mika.valden@tuni.fi harri.ali-loytty@tuni.fi; Tampere Microscopy Center, Tampere University P.O. Box 692 FI-33014 Tampere Finland

## Abstract

Bimetallic Ag–Au/TiO_2_ nanocomposites were synthesized by sequential photodeposition in order to investigate the effect of surface plasmon resonance (SPR) properties on photocatalytic activity for solar water splitting and methylene blue (MB) degradation. The photodeposition times were optimized for monometallic Ag/TiO_2_ and Au/TiO_2_ nanocomposites to yield maximum SPR absorption in the visible range. It was found that the photocatalytic activity of bimetallic Ag–Au/TiO_2_ nanocomposites outperformed monometallic nanocomposites only when Au was photodeposited first on TiO_2_, which was attributed to Au-core–Ag-shell nanoparticle morphology. In contrast, reversing the photodeposition order resulted in Ag–Au alloy nanoparticle morphology, which was mediated by the galvanic replacement reaction during the second photodeposition. Alloying was not beneficial to the photocatalytic activity. These results demonstrate alloying during sequential photodeposition providing new insights for the synthesis of TiO_2_-based photocatalysts with plasmon-enhanced absorption in the visible range.

## Abstract 2

Plasmonic AgAu *alloy* nanoparticles were fabricated on TiO_2_ (P25) utilizing the galvanic replacement reaction during sequential photodeposition of Au after Ag.

## Graphical abstract

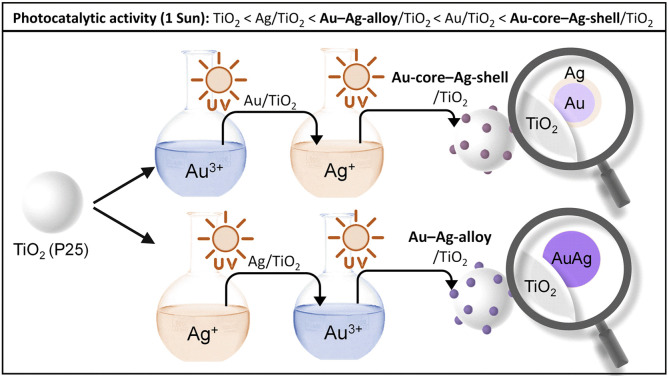

## Introduction

Considering the future perspective, photocatalytic energy production serves as the ultimate pathway to tackle the crises of energy and global warming. Although a wide range of materials are found appropriate for photocatalytic applications, titanium dioxide (TiO_2_) is still the most investigated benchmark since the discovery and demonstration of photocatalytic water splitting by Fujishima and Honda in 1972.^1^ TiO_2_ being nontoxic, thermally stable, highly active and cost effective has been studied immensely due to its promising applications in catalysis, wastewater treatments and harvesting solar light.^2–6^ However, the large band gap energies of 3.0 eV for the rutile phase and 3.2 eV for the anatase phase TiO_2_ limit the light absorption to the UV range.^7,8^ Hence, one approach to improve the photocatalytic efficiency of TiO_2_ is to extend the light harvesting capability of TiO_2_ to the visible region. The activity of a bare TiO_2_ photocatalyst is often limited by the high recombination rate at defects at the surface and within the lattice.^9,10^

**Fig. 1:**
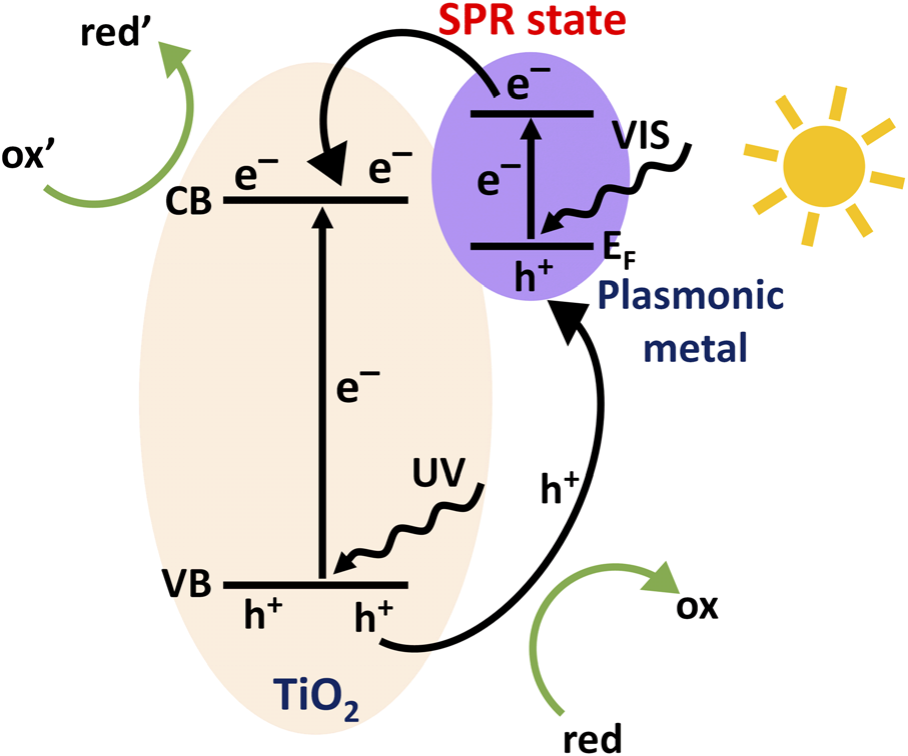
Schematic illustration of the charge transfer processes within a TiO_2_ photocatalyst loaded with plasmonic metal nanoparticles.

To overcome these impediments, numerous strategies have been employed so far including metal and non-metal doping as well as functionalizing the TiO_2_ surface.^11–17^ In recent years, the deposition of noble metal nanoparticles (*e.g.*, Au, Ag, Pd and Pt) on TiO_2_ has acquired strong attention for providing means to increase both, solar light absorption in the visible range and electron–hole pair separation which both result in increased photocatalytic activity.^18–22^ These metal nanoparticles introduce extra energy levels into the wide band gap of TiO_2_ that affect the charge transfer properties.^23–25^ The Schottky junction that forms at the metal–TiO_2_ interface can act as a trap for photo-generated electrons preventing the detrimental recombination. In addition, these metallic nanoparticles provide plasmonic enhancement by hot electron transfer and the plasmon induced resonant energy transfer (PIRET) process.^19^ Charge transfer processes within a TiO_2_ photocatalyst loaded with plasmonic metal nanoparticles are schematically depicted in Fig. 1. Electron–hole pairs are formed within TiO_2_ by the UV part of the solar spectrum, while the absorption of visible light excites electrons from the Fermi-edge of metallic nanoparticles to the surface plasmon resonance (SPR) state. For a given photocatalytic process, photoexcited electrons drive reduction reactions (ox′ + e^−^ → red′) while holes drive oxidation reactions (red + h^+^ → ox) at the catalyst surface.

The plasmonic enhancement depends strongly on the electronic structure and size of the metal nanoparticle, which allows for a wide range of optimization strategies for a tailored photocatalyst. Due to their optimal SPR absorption peak positions in the visible range, silver and gold nanoparticles are the most studied plasmonic nanoparticles in artificial photosynthesis.^19–21,23,24,26^ Fabrication of plasmonic nanoparticles involving two or more metals on TiO_2_ is considered a promising approach to further improve the photocatalytic activity but optimization of the fabrication process is far from trivial due to the complex structure–performance relationship of plasmonic photocatalyst nanoparticles.^27–29^

Different methods have been applied to deposit plasmonic metal nanoparticles on the TiO_2_ surface including electrodeposition,^30,31^ atomic layer deposition,^32^ sputtering,^33^ flame spray pyrolysis,^26,34^ deposition precipitation,^35,36^ impregnation,^30^ physical mixing,^37^ coprecipitation^38^ and photodeposition.^39,40^ However, compared to other methods, photodeposition is an appealing method since it does not require elevated temperatures, it is suitable for particulate photocatalysts, and most importantly, the growth process is directed by the photo-induced reduction of metal ions from the solution phase to the semiconductor particle surface. Plasmonic nanoparticles of different metals can be grown sequentially by photodeposition but a different growth process is expected if the semiconductor already has plasmonic nanoparticles. It was recently shown that the photodeposition of Ag on TiO_2_ with plasmonic Au nanoparticles resulted in a Au-core–Ag-shell structure that exhibited increased efficiency for photocatalytic hydrogen production.^29^

In this work, we investigate the effect of the photodeposition sequence on the photocatalytic activity of plasmonic Ag–Au/TiO_2_ particulate photocatalysts. We show that the photodeposition of Ag before Au results in worse photocatalytic activity compared to the champion photocatalyst for which Ag was photodeposited after Au. The superior performance is assigned to the Au-core–Ag-shell structure with an optimal electronic structure, which can only form when Au is photodeposited first. Reversing the photodeposition order results in Ag–Au alloy formation that is rationalized by the galvanic replacement reaction (3Ag + Au^3+^ → Au + 3Ag^+^)^41,42^ taking place during the second photodeposition. Thus, the work demonstrates a new synthesis strategy for plasmonic Ag–Au alloy nanoparticles.

## Experimental section

### Materials

Gold chloride (HAuCl_4_·3H_2_O, Sigma Aldrich, ≥99.9% trace metals basis), silver nitrate (AgNO_3_, Sigma Aldrich, BioXtra >99% titration), TiO_2_ (Degussa P25, Sigma Aldrich, Aeroxide® P25, ≥99.5% trace metals basis, particle size approx. 21 nm using TEM analysis and specific surface area 35–65 m^2^ g^−1^), methanol (VWR, ≥99.8% ACS reagent), methylene blue (MB) (Sigma Aldrich, 0.05 wt% in H_2_O) and deionized (DI) water from an ultrafiltration system were used in the work. All the chemicals were used without any further purification.

### Synthesis of mono-metallic Au/TiO_2_ and Ag/TiO_2_ nanocomposites

Synthesis of nanocomposite photocatalyst particles was adapted from ref. 29 in which the metal loadings in the photodeposition solutions were optimized to yield maximum solar water splitting activity for Au/TiO_2_ and Ag–Au/TiO_2_. Au/TiO_2_ catalyst particles were synthesized by the photodeposition method. 0.001 M stock solution of gold chloride was prepared in DI water. 0.5 g TiO_2_ was taken into a 500 mL quartz round bottom flask (RBF) and mixed with 100 mL methanol and 30 mL DI water. 0.25 wt% of Au from the precursor stock solution was added to this suspension. The reactor containing the suspension of TiO_2_ was sealed with a rubber septum following purging with nitrogen gas for 30 min by penetrating a long needle inside the suspension through the rubber septum. Another needle was inserted, through the rubber septum inside the headspace of the reactor to remove O_2_ and other gases from the reactor. After purging, the suspension containing TiO_2_ nanoparticles and the Au precursor was illuminated under a UV light source (200 W ELC-800 UV lamp, high pressure 200 Watt DC short arc mercury vapor lamp). To optimize the photodeposition time, a series of samples was prepared by varying the photodeposition time between 10 min and 80 min. The color of the HAuCl_4_–TiO_2_ suspension changed from light yellow to violet-pink upon UV light irradiation confirming the metal deposition on TiO_2_. The Au/TiO_2_ catalyst particles were then recovered by centrifugation at 8000 RPM (Z 233 M, Hermle LaborTechnik GmbH) for 15 min and drying overnight in an oven at 70 °C. Similarly, Ag/TiO_2_ catalyst particles were prepared by following the procedure mentioned above by adding 0.14 wt% of Ag from 0.001 M AgNO_3_ stock solution to a water–methanol–TiO_2_ mixture. The color of the AgNO_3_–TiO_2_ suspension changed from white to light orange upon UV light irradiation.

### Synthesis of bimetallic Ag–Au/TiO_2_ and Au–Ag/TiO_2_ nanocomposites

0.5 g Au/TiO_2_ nanoparticles that resulted from the 40 min photodeposition time was taken into a 500 mL quartz RBF followed by the addition of 100 mL methanol and 30 mL DI water. The solution was then sonicated for 30 min to obtain a well-dispersed suspension of Au/TiO_2_ nanoparticles in methanol and water. After sonication, 0.14 wt% of Ag was added into the suspension from the 0.001 M AgNO_3_ stock solution. The suspension was then illuminated under UV light for 20 min for the photodeposition of Ag on Au/TiO_2_. The suspension was then centrifuged for 15 min at 8000 RPM and dried overnight in an oven at 70 °C to recover bimetallic Ag–Au/TiO_2_ nanocomposite particles. The Au–Ag/TiO_2_ nanocomposite was synthesized in a similar way by taking 0.5 g Ag/TiO_2_ nanoparticles that resulted from a 20 min photodeposition time, adding 0.25 wt% Au from 0.001 M HAuCl_4_ stock solution and using 40 min as the photodeposition time. The nominal molar Au : Ag ratio of bimetallic samples was 1 : 1.02.

### Characterization of nanoparticles

For the characterization of nanoparticle properties, thin film samples were prepared *via* drop-casting a water-powder mixture onto a microscopy glass and drying in an oven at 70 °C overnight. The optical properties of the catalyst nanoparticles were studied using a spectrophotometer equipped with an integrating sphere detector (PerkinElmer®LAMBDA 1050 UV/Vis/NIR). Diffuse reflectance spectra were recorded from 300 nm to 750 nm. The metal loading on catalyst nanocomposites was analyzed by inductively coupled plasma mass spectrometry (ICP-MS, Thermo Scientific iCAP™ RQ). For the ICP-MS analysis, Ag and Au were first extracted from weighed powder samples with concentrated aqua regia (3 : 1, v/v, HCl–HNO_3_) and then diluted appropriately for the measurement. XPS measurements were carried out in an ultra-high vacuum system equipped with an electron spectrometer (V. G. Microtech, CLAM4 MCD LNo5) using Al Kα (*hν* = 1486.6 eV) excitation and a constant pass energy of 100 eV. The data was analyzed using Casa XPS software.^43^ The binding energy scale was calibrated according to C 1s (C–C/H) at 285.0 eV. A scanning transmission electron microscope (S/TEM, Jeol JEM-F200, JEOL Ltd., Tokyo, Japan) equipped with an energy dispersive spectrometer (EDS, Jeol Dual EDS for F200, JEOL Ltd., Tokyo, Japan), was used to study the morphology and elemental composition of nanoparticles. The Raman spectra of the samples were measured using a Renishaw inVia Qontor Raman microscope and the measurements were performed using a 532 nm laser.

### Photocatalytic tests

The photocatalytic performance of catalyst particles was tested using two model reactions; the photocatalytic hydrogen production and the photocatalytic degradation of methylene blue.

For the hydrogen production reaction, 20 mg of photocatalyst nanoparticles was taken into a 50 mL quartz round bottom (QRB) photoreactor and mixed with 40 mL of 25% v/v aqueous methanol solution and sealed with a rubber septum. In the reaction, methanol acts as a hole scavenger.^44^ A solar simulator (HAL-C100, Asahi Spectra Co., Ltd.) was used as the light source and the light intensity was fixed at 1 sun conditions using a 1 sun checker (model CS-30, Asahi Spectra Co., Ltd.). Before beginning the test, the slurry was purged with nitrogen gas for 20 min to remove dissolved hydrogen/oxygen from the slurry and from the headspace of the reactor. During the test, the slurry was continuously stirred using a magnetic stirrer. The photoreactor was irradiated for 3 hours in total while the headspace was sampled every 30 min by ejecting a 500 μL gas sample through the rubber septum using a gas-tight syringe. The amount of hydrogen was analyzed using a Thermo Scientific Trace 1310 gas chromatograph (GC) equipped with a headspace injector, a TG-Bond Msieve 5A column (length: 30 m, internal diameter: 0.53 mm) and a thermal conductivity detector (TCD). The detector inlet temperature and filament temperature were kept at 250 °C and 350 °C, respectively. Nitrogen was used as the carrier gas. The calibration curve for hydrogen was obtained by the injection of a volume series of hydrogen (99.9999%, Oy AGA Ab) into the GC. For each photocatalyst the test was repeated three times to confirm the reproducibility of the photocatalytic activity.

For the methylene blue photodegradation test, a 100 ppm photocatalyst was mixed with 50 mL deionized water followed by the addition of 2 ppm methylene blue (MB) solution (Sigma Aldrich, 0.05 wt% in H_2_O) in the QRB reactor. In order to establish MB adsorption equilibrium with the catalyst, the slurry was first stirred in the dark for 90 min. Then, a 3 mL sample was taken out of the reactor as the initial concentration (*C*_0_) of MB before the illumination was started with the solar simulator under 1 sun conditions. During the experiment, a 3 mL sample was drawn out of the reactor every 10 min for 50 min. Then, centrifugation was performed for all the samples at 6500 RPM for 15 min to separate the catalyst from the MB solution. Finally, the centrifuged solutions were decanted into acrylic cuvettes with a light path length of 10 mm for absorbance measurement. The concentration of MB in solution was analyzed based on MB absorption at 635 nm using a calibration curve measured for a range of MB concentrations. The home-made test setup consisted of a cuvette holder (ThorLabs CVH100), a laser source (ThorLabs CPS635R, collimated laser diode module, 1.2 mW, 635 nm), a photodetector (ThorLabs SM1PD1A, silicon photodiode, detection range 350–1100 nm) and a photodiode amplifier (ThorLabs PDA200C).^45^

## Results and discussion

**Fig. 2:**
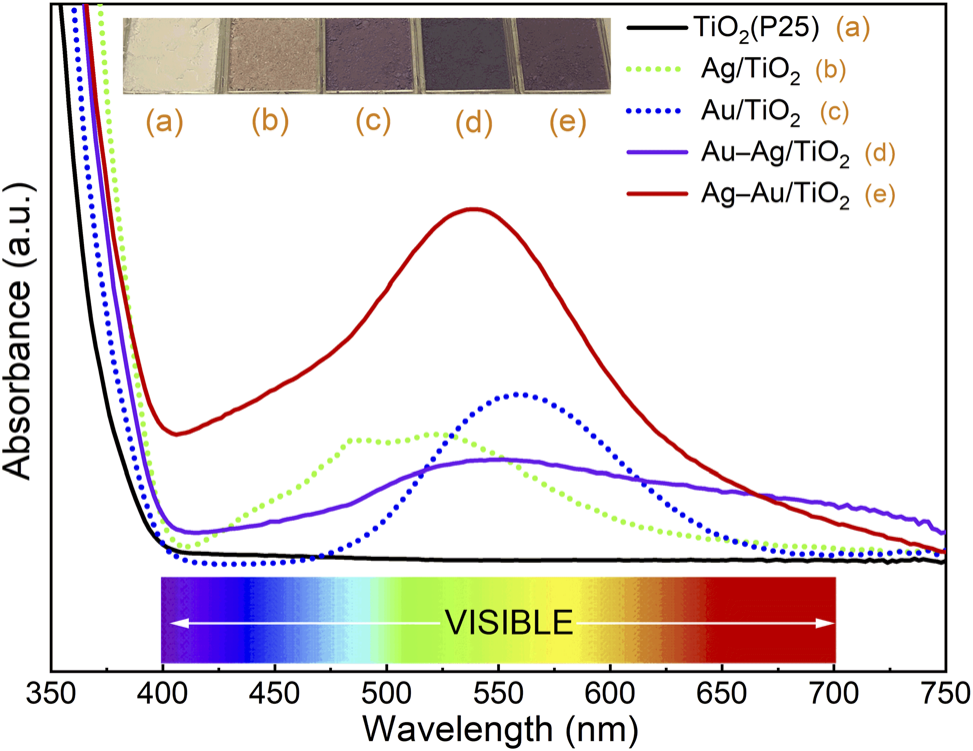
UV/Vis absorption spectra and pictures of (a) TiO_2_ (P25), (b) Ag/TiO_2_, (c) Au/TiO_2_, (d) Au–Ag/TiO_2_, and (e) Ag–Au/TiO_2_.

First, photodeposition times for mono-metallic Ag/TiO_2_ and Au/TiO_2_ nanocomposites were optimized to yield maximum SPR peak intensities at 515 nm and 555 nm, respectively (Fig. S1†).^46,47^ The maximum SPR peak intensities were obtained by using 20 min and 40 min photodeposition times for Ag and Au, respectively. Then, bimetallic samples were prepared by sequential photodeposition of Au and Ag using the photodeposition times optimized for mono-metallic samples. The UV-Vis absorption spectra of different samples are depicted in Fig. 2.

The strong absorbance threshold at 400 nm (*i.e.*, in the ultraviolet region) is attributed to the bandgap absorption of TiO_2_.^20^ No absorbance throughout the visible range (400–800 nm) was observed for TiO_2_ (P25). The absorbance of bimetallic nanocomposites covers a wider range throughout the visible region (400–750 nm) compared to the mono-metallic samples. This indicates the extensive absorption capacity of the bimetallic nanocomposites over the bare TiO_2_ and monometallic nanocomposites. The deposition order had a strong effect on the absorbance of bimetallic nanocomposites. The sample for which Au was deposited first (Ag–Au/TiO_2_) shows maximum SPR band intensity at 535 nm, between the SPR peaks of Ag and Au, and much stronger absorbance in the visual range compared to the sample for which Ag was deposited first (Au–Ag/TiO_2_). This evidences that the plasmonic metal on TiO_2_ has a strong influence on the photodeposition of the second metal.

The structural features of samples were analyzed by Raman spectroscopy. As shown in Fig. S2,† only peaks corresponding to anatase TiO_2_ were observed for all the samples. Additionally, strong enhancements in Raman signal intensities were observed for all metal/TiO_2_ samples compared to plain TiO_2_ supporting successful integration of plasmonic nanoparticles.

Table 1 depicts Ag and Au loading in the samples. The Ag loading was 0.14–0.15 wt% and Au loading was 0.29–0.39 wt% for all the samples. The bimetallic samples had similar metal loadings to the monometallic samples. The Ag and Au concentrations were close to the concentrations in the stock solutions indicating high yield for the photodeposition in all the samples.

**Table 1:** Ag and Au loadings in the samples

Wt%	Ag	Au
TiO_2_	0.00	0.00
Ag/TiO_2_	0.14	0.00
Au/TiO_2_	0.00	0.29
Au–Ag/TiO_2_	0.15	0.39
Ag–Au/TiO_2_	0.14	0.28

**Fig. 3:**
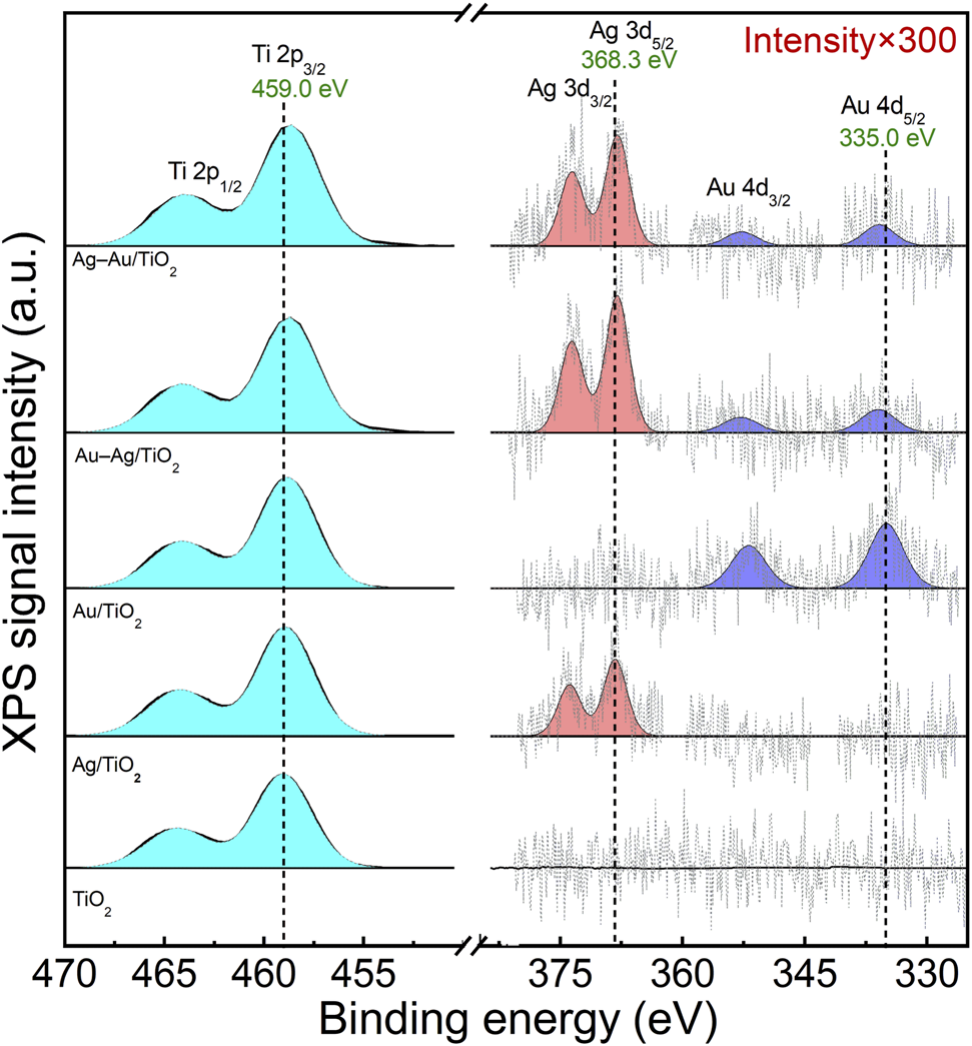
XPS spectra of Ti 2p, Ag 3d and Au 4d peaks for TiO_2_ (P25), Ag/TiO_2_, Au/TiO_2_, Au–Ag/TiO_2_, and Ag–Au/TiO_2_.

The chemical states of elements were analyzed by X-ray photoelectron spectroscopy (XPS). Fig. 3 shows spectral regions for Ti 2p, Ag 3d and Au 4d doublet transitions. The strong Ti 2p_3/2_ at 459.0 eV corresponds to Ti^4+^ in TiO_2_.^48^ The small Ag and Au loadings resulted in XPS signals that were close to the detection limit: Ag 3d_5/2_ at 368.3 eV and Au 4d_5/2_ at 335.0 eV correspond to metallic Ag and Au, respectively.^48^ Due to the low metal loading no difference in the chemical states of Ag and Au was able to be reliably differentiated between the mono and bimetallic samples. Thus, XPS analysis suggests the reduction of Ag and Au ions upon the photodeposition process in all the samples. The presence of Au and Ag in metallic form supports the plasmonic enhancement of visible light absorption.

**Fig. 4:**
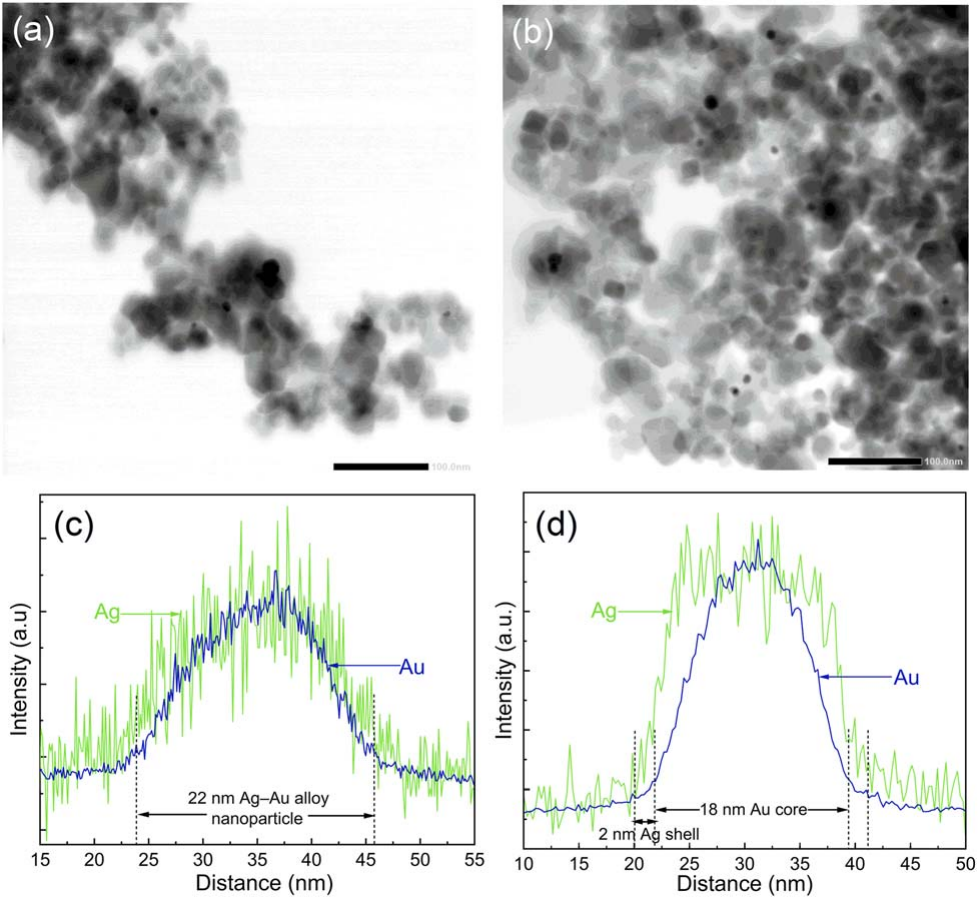
Bright field STEM images of (a) Au–Ag/TiO_2_ and (b) Ag–Au/TiO_2_. STEM-EDS line analysis for representative bimetallic nanoparticles on (c) Au–Ag/TiO_2_ and (d) Ag–Au/TiO_2_. The scale bar in (a) and (b) is 100 nm.

In order to analyze the morphology of bimetallic nanoparticles, STEM-EDS analysis was performed and presented in Fig. 4. The bright field STEM images in Fig. 4a and b for Ag–Au/TiO_2_ and Au–Ag/TiO_2_ show a similar sparse distribution of metallic nanoparticles on TiO_2_ agglomerates for both samples. However, the STEM-EDS line analysis shown in Fig. 4c and d reveals the striking difference between the nanoparticle morphologies (*cf.*, Fig. S5 and S6† for STEM-EDS line analysis of more particles). In the case of Ag–Au/TiO_2_, the Au signal is detected only in the core (18 nm in diameter) of the nanoparticles and the Ag signal extends to the surface of nanoparticles forming a 2 nm thick shell. The result indicates Au-core–Ag-shell morphology for the Ag–Au/TiO_2_ NPs. In contrast, Ag and Au signals are evenly distributed on the Au–Ag/TiO_2_ sample suggesting Ag–Au alloy NP morphology. Surprisingly, Ag–Au alloy formation was observed despite the sequential photodeposition.

Based on thermodynamics, the formation of Au NPs is considered the most likely.^49^ The cohesive energies of core–shell and alloy Ag–Au NPs are between pure Ag and Au NPs, and alloy NPs are more stable than core–shell structures. Thus, the only rational explanation for our finding is that the least stable Ag NPs react with Au during subsequent Au photodeposition forming Ag–Au alloy NPs.

Alloy NP formation during the reduction process requires metal diffusion rates to be high compared to the reduction rates. Obtaining a thermodynamically most stable phase often requires heating. Indeed, Lasserus *et al.* reported alloying of both Ag@Au and Au@Ag core–shell nanoparticles of 2 nm radius to onset at 500–550 K.^50^ Au–Ag alloy NP synthesis has been previously reported for co-reduction of Au and Ag salts^51^ but, to the best of our knowledge, not as a result of sequential photoreduction. A plausible explanation for the alloy formation would be plasmonic heating induced mixing of Ag with Au during Au photodeposition^52^ but to induce a sufficient temperature increase would require much stronger excitation power, *e.g.*, by a laser source at the SPR energy.^53^ Instead, considering the electrochemical potential difference between Ag and Au provides another feasible explanation for the alloy formation that can proceed at room temperature, namely the galvanic replacement reaction. In the galvanic replacement reaction, less stable Ag oxidizes with Au^3+^ reduction (3Ag + Au^3+^ → Au + 3Ag^+^) which has been utilized previously in the fabrication of Au nanocages using sacrificial Ag nanostructures.^42^ However, under the photodeposition conditions any dissolved Ag^+^ is subject to photoreduction that effectively results in the observed Ag–Au alloy NP formation.

**Fig. 5:**
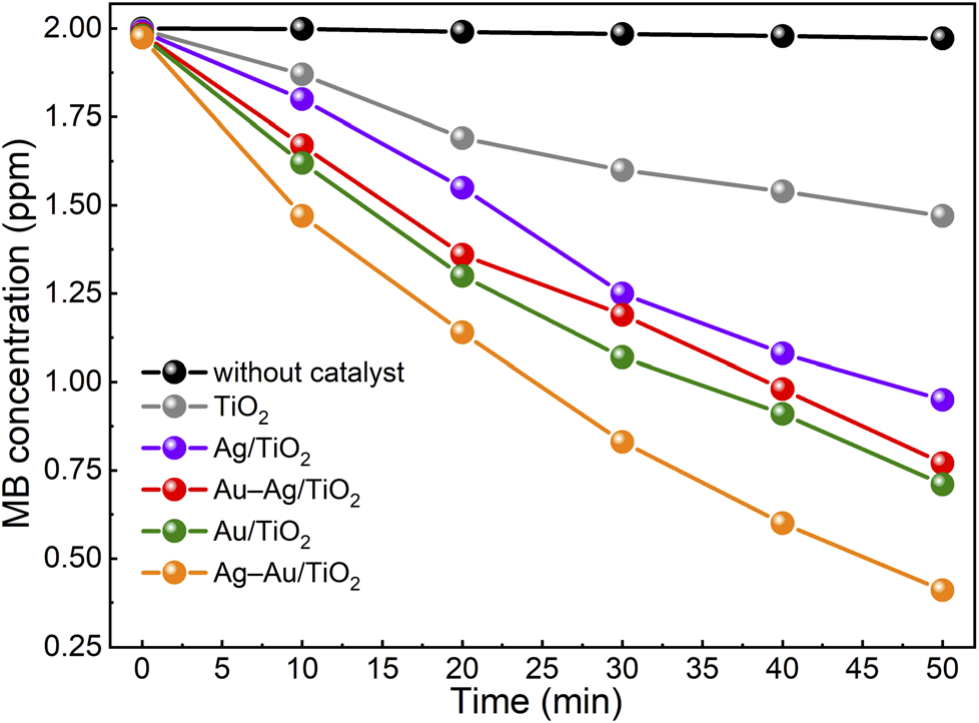
Photocatalytic methylene blue degradation under solar irradiation in the presence of catalyst samples.

Fig. 5 shows the activity of samples for photocatalytic degradation of methylene blue. The degradation kinetics was found to follow the first order reaction kinetics (Fig. S3†), *c* = *c*_0_ e^−*κt*^, with apparent rate constant values, *κ*, presented in Table 2. The activity was found to increase in the order: TiO_2_ < Ag/TiO_2_ < Au–Ag/TiO_2_ < Au/TiO_2_ < Ag–Au/TiO_2_. The MB test was performed without any catalyst to determine the photolysis rate of MB under the test conditions. The photolysis rate was found to be small (∼1%) compared to the degradation rate recorded with the champion photocatalyst. During the preconditioning in the dark, the MB concentration decreased only a little from 2 ppm to 1.974–1.996 ppm as a result of either MB adsorption on catalysts or activity in the dark. The color of catalyst particles turned bluish in the dark confirming MB adsorption. Possible catalyst activity in the dark towards MB degradation was considered insignificant compared to the measured photoactivities.

**Table 2:** Apparent rate constant (*κ*_app_) values for methylene blue photodegradation under 1 sun conditions

Catalyst	*κ*_app_ (min^−1^)
No catalyst	0.0003
TiO_2_	0.0062
Ag/TiO_2_	0.0149
Au–Ag/TiO_2_	0.0191
Au/TiO_2_	0.0205
Ag–Au/TiO_2_	0.0306

The Ag–Au/TiO_2_ catalyst was the most active catalyst depicting 390% higher activity for photocatalytic MB degradation compared to the bare TiO_2_ photocatalyst standard and 49% higher activity compared to Au/TiO_2_. The activities were found to have a good correlation with visible light absorption as shown in Fig. 2. It is worth noticing that monometallic Au/TiO_2_ depicted higher activity compared to bimetallic Au–Ag/TiO_2_.

**Fig. 6:**
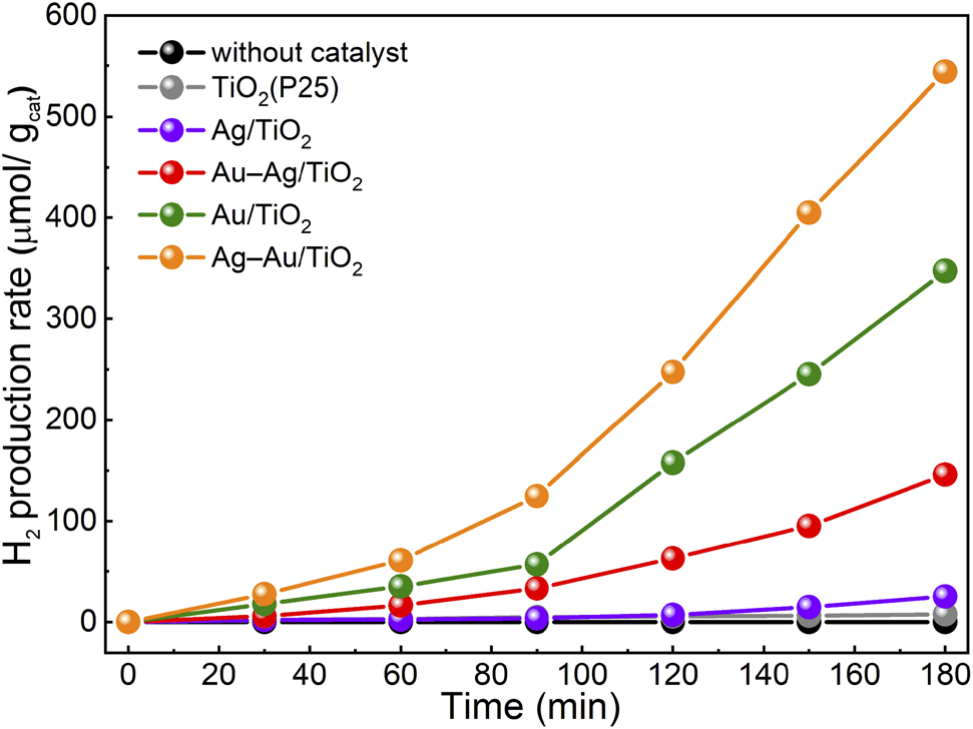
Photocatalytic H_2_ production from aqueous methanol solution under solar irradiation in the presence of catalyst samples.

The activity of samples for photocatalytic hydrogen production from aqueous methanol solution was tested. The hydrogen production yields for samples during the 3 h test are presented in Fig. 6. All the samples showed an induction period of ∼90 min after which the H_2_ yield increased and followed a linear trend. Such an induction period has been proposed to result from adsorbed carbon impurities that are less effective hole scavengers than methanol and are consumed first.^54^ The H_2_ production rates after the induction period are shown in Table 3. The H_2_ production rate was found to be drastically higher for the samples with NPs containing Au compared to plain TiO_2_ or Ag/TiO_2_. The activity of plain TiO_2_ was found to be insignificant. The activities for H_2_ production were found to increase in the same order that was observed in the methylene blue degradation test: TiO_2_ < Ag/TiO_2_ < Au–Ag/TiO_2_ < Au/TiO_2_ < Ag–Au/TiO_2_. However, the differences between the samples were stronger. Specifically, the activity of monometallic Au/TiO_2_ for H_2_ production was clearly stronger than that of Au–Ag/TiO_2_. The reproducibility of the photocatalytic hydrogen production activity was found to be high when the test was repeated three times using a fresh sample (Fig. S4†).

**Table 3:** H_2_ evolution rate in aqueous methanol solution under 1 sun conditions

Catalyst	H_2_ rate (μmol h^−1^ g^−1^)
No catalyst	0
TiO_2_	2
Ag/TiO_2_	14
Au–Ag/TiO_2_	74
Au/TiO_2_	190
Ag–Au/TiO_2_	280

The highest activity in both tests was obtained for Ag–Au/TiO_2_, *i.e.*, the Au-core–Ag-shell structure. The obtained H_2_ production rate was smaller (280 *vs.* 718 μmol h^−1^ g^−1^)^29^ compared to a previously reported value for similar synthesis and test procedure. An even stronger difference was found for Ag/TiO_2_ (14 *vs.* 355 μmol h^−1^ g^−1^) that demonstrates the challenge in the comparison of the results between two independent works. Nevertheless, the order of activities was the same (TiO_2_ < Ag/TiO_2_ < Au/TiO_2_ < Ag(shell)–Au(core)/TiO_2_). Interestingly, no significant differences were observed in either chemical composition or metal loading between bimetallic Ag–Au/TiO_2_ and Au–Ag/TiO_2_ samples. In contrast, the STEM results of Au–Ag/TiO_2_ evidenced Au–Ag alloy nanoparticle morphology that was found less beneficial to visible light absorption and photocatalytic activity. Therefore, the differences can be assigned to the morphology that was induced by the photodeposition sequence.

The activity of monometallic Au/TiO_2_ exceeded that of alloy Au–Ag/TiO_2_ in both tests. This result is consistent with that by Chiarello *et al.* who found photocatalytic activities to increase in the order of TiO_2_ < Ag/TiO_2_ < alloy Au–Ag/TiO_2_ < Au/TiO_2_ (ref. 26) but in apparent contradiction with recent reports by Haider *et al.* and Malik *et al.* where Au–Ag alloy NPs on TiO_2_ have shown higher photocatalytic activities compared to monometallic NPs.^55,56^

In both works, however, the experimental conditions were different and focussed on the water oxidation reaction, which challenges comparison of the results. Also, the synthesis of catalysts was different. Haider *et al.* synthesized plasmonic NPs separately whereas in our photodeposition process the growth of NPs was inherently directed to the active sites of TiO_2_ where electron transfer from TiO_2_ is favoured.

Both Ag and Au NPs induced visible light absorption to a similar extent but Au NPs resulted in significantly higher photocatalytic activities. This difference in the photocatalytic performance can be attributed to the higher Schottky barrier at the Au/TiO_2_ interface compared to the Ag/TiO_2_ interface. The work function values for different facets of Ag and Au are between 4.64–4.74 eV and 5.31–5.47 eV, respectively^57^ whereas the work function value for TiO_2_ is 4.6–4.7 eV.^58^ The higher work function difference between the metal and semiconductor indicates a higher Schottky barrier and therefore better performance as a photocatalyst.^26^ The superior performance of the Ag–Au/TiO_2_ photocatalyst is therefore the result of the combined effects of plasmonic enhancement of visible light absorption by the Au-core–Ag-shell structure and favourable charge carrier separation at the Au/TiO_2_ interface.

The activities of catalysts in the two tests increased in the same order but the relative activities were different. Most notably, while plain TiO_2_ showed reasonable activity towards MB photodegradation, the activity towards H_2_ production was minute, despite the presence of methanol as the hole scavenger. The differences in the relative activities evidence the difference in the involved surface reactions. In the MB test, organic MB molecules are mineralized into inorganic products in a photooxidative process with the reduction of dissolved oxygen.^59^ In the H_2_ production test, the photocatalytic water splitting reaction proceeds in anaerobic aqueous methanol solution where methanol serves as the hole scavenger and oxidizes more easily than water. The photocatalytic H_2_ production results from either the water or methanol reduction reaction.^60^ Metallic nanoparticles on TiO_2_ facilitate the separation of photoinduced charge carriers in both tests but show more dramatic improvements in activity in the H_2_ production test. Conversely, photodegradation of MB is a more facile reaction as suggested by the reasonably high reaction rate of plain TiO_2_.

Our results show that the plasmonic Au-core–Ag-shell structure on TiO_2_ outperforms the Au–Ag alloy structure in terms of photocatalytic activity. A potential concern with the Ag-shell is, however, its long-term stability since Ag NPs are susceptible to photoanodic dissolution and cathodic re-deposition.^61^ It is suggested that the Au-core may improve the stability of the Ag-shell by directing the re-deposition of dissolved Ag^+^. Alloying Ag with Au has been suggested as an alternative approach for improving the stability^55^ and our results demonstrate a new means to synthesize Au–Ag alloy NPs on TiO_2_ utilizing the galvanic replacement reaction during sequential photodeposition.

## Conclusions

In this work, the influence of the photodeposition sequence on the photocatalytic activity of plasmonic bimetallic Ag–Au/TiO_2_ nanocomposites was studied. The absorption of TiO_2_ in the visible range was successfully increased by plasmonic mono- and bimetallic nanoparticles. Photodeposition of Au before Ag resulted in a previously reported Ag-shell–Au-core-structure. In contrast, reversing the photodeposition order resulted in unexpected Au–Ag-alloy nanoparticle morphology that was rationalized by the galvanic replacement reaction (3Ag + Au^3+^ → Au + 3Ag^+^) during the second photodeposition.

The photocatalytic activities were tested under 1 sun conditions in terms of methylene blue photodegradation and H_2_ production from aqueous methanol solution. The activities were found to increase in the same order in the two tests: TiO_2_ < Ag/TiO_2_ < Au–Ag-alloy/TiO_2_ < Au/TiO_2_ < Ag-shell–Au-core/TiO_2_. Thus, our results show that the plasmonic Au-core–Ag-shell structure outperforms the Au–Ag alloy structure on TiO_2_ in terms of photocatalytic activity. These results challenge some recent studies showing exceptionally high activity of Au–Ag alloy NPs on TiO_2_ towards photocatalytic water oxidation compared to monometallic Ag and Au.

The demonstrated galvanic replacement reaction mediated alloying mechanism during sequential photodeposition provides new insights for the synthesis of TiO_2_-based photocatalysts with plasmon-enhanced absorption in the visible range. On one hand, it can be seen as a degradation mechanism during core–shell nanoparticle synthesis or during photocatalyst operation. On the other hand, it can be utilized for the synthesis of plasmonic alloy nanoparticles as opposed to conventional co-reduction or synthesis involving post deposition heat treatment.

## Conflicts of interest

There are no conflicts to declare.

## Supplementary Material

NA-004-D2NA00440B-s001
