# Supplementary material for: Influence of the photodeposition sequence on the photocatalytic activity of plasmonic Ag–Au/TiO2 nanocomposites
Source: Nanoscale Adv. 2022 Aug 31;4(20):4335–43. doi: 10.1039/d2na00440b (PMC9552921; doi:10.1039/d2na00440b)
Supplement: NA-004-D2NA00440B-s001 [file NA-004-D2NA00440B-s001.pdf]

Electronic Supplementary Material (ESI) for Nanoscale Advances.
This journal is © The Royal Society of Chemistry 2022

Electronic Supplementary Information

Influence of Photodeposition Sequence on the Photocatalytic Activity of Plasmonic Ag–Au/TiO_2 Nanocomposites

Bela D. Bhuskute[a], Harri Ali-Löytty*[,a], Mari Honkanen[b], Turkka Salminen[b], and Mika Valden*[,a]

[a]*Surface Science Group, Photonics Laboratory, P.O. Box 692, FI-33014 Tampere University, Finland.*

[b]*Tampere Microscopy Center, Tampere University, P.O. Box 692, FI-33014 Tampere University, Finland Address here.*

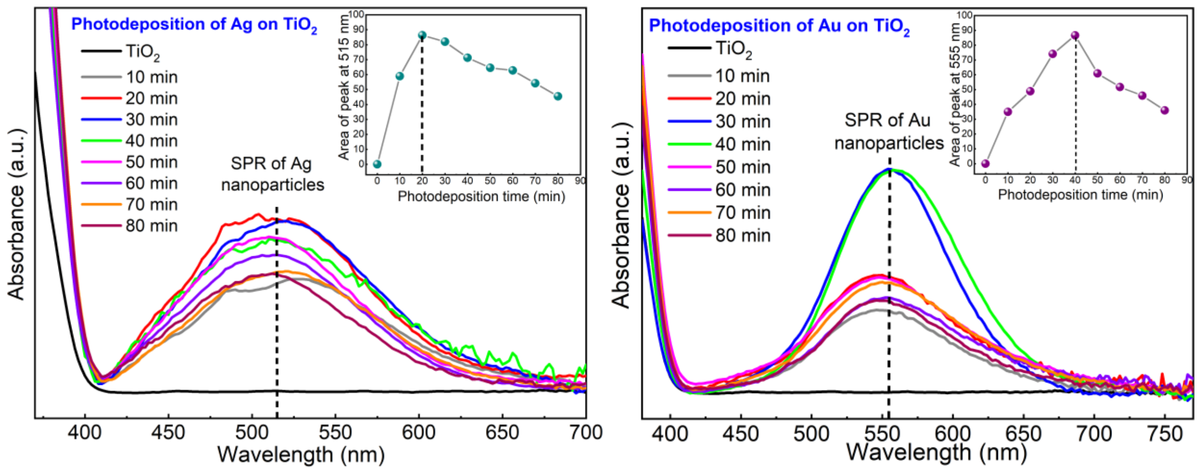

Figure S1. UV-Vis absorption spectra of Ag/TiO_2 and Au/TiO_2 with respect to photodeposition time.

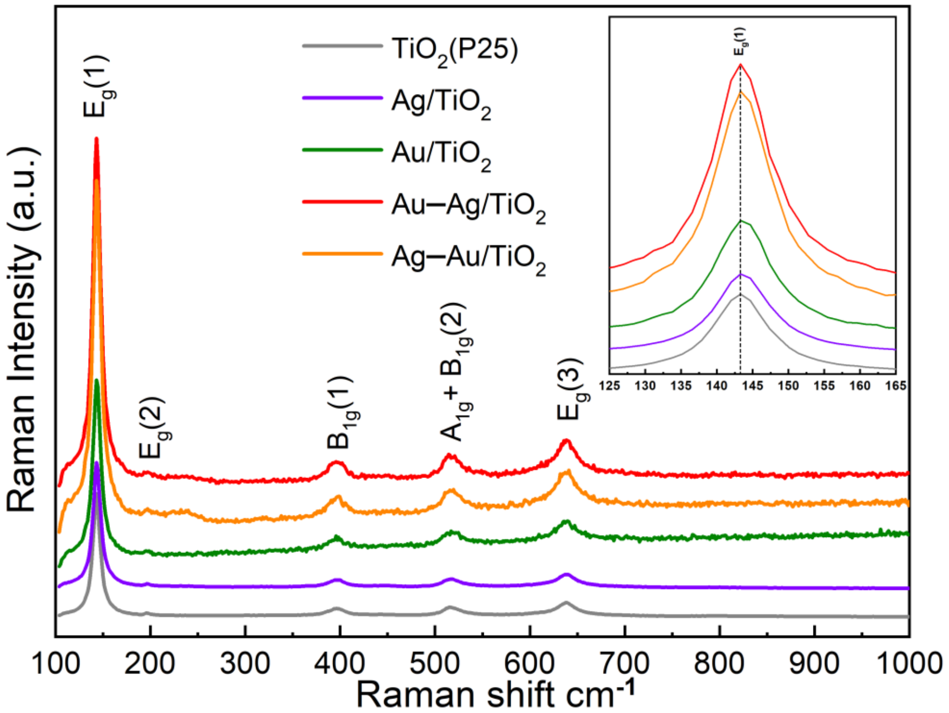

Figure S2. Normalized Raman spectra of synthesized catalysts showing structural features and active vibration modes of only anatase TiO_2. Peaks at 147, 200, 398, 515 and 639 cm^{-1} corresponding to E_g (1), E_g (2), B_{1g} (1), A_{1g} + B_{1g} (2) and E_g (3) active vibration modes, respectively.

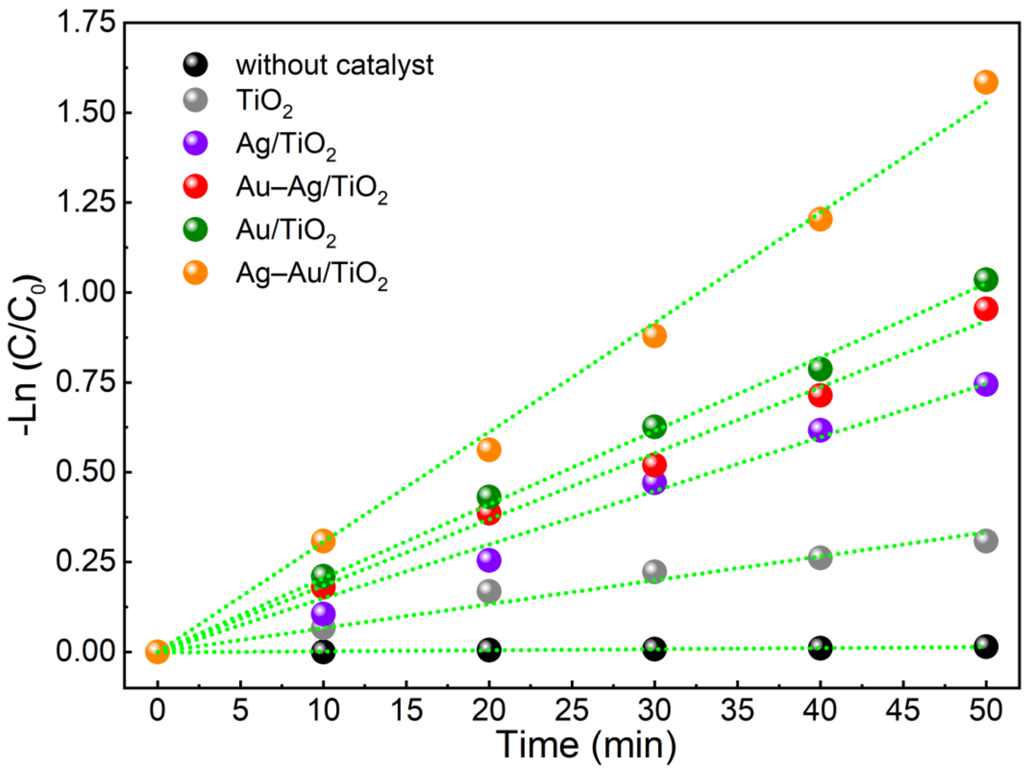

Figure S3. Methylene blue degradation kinetics under solar irradiation.

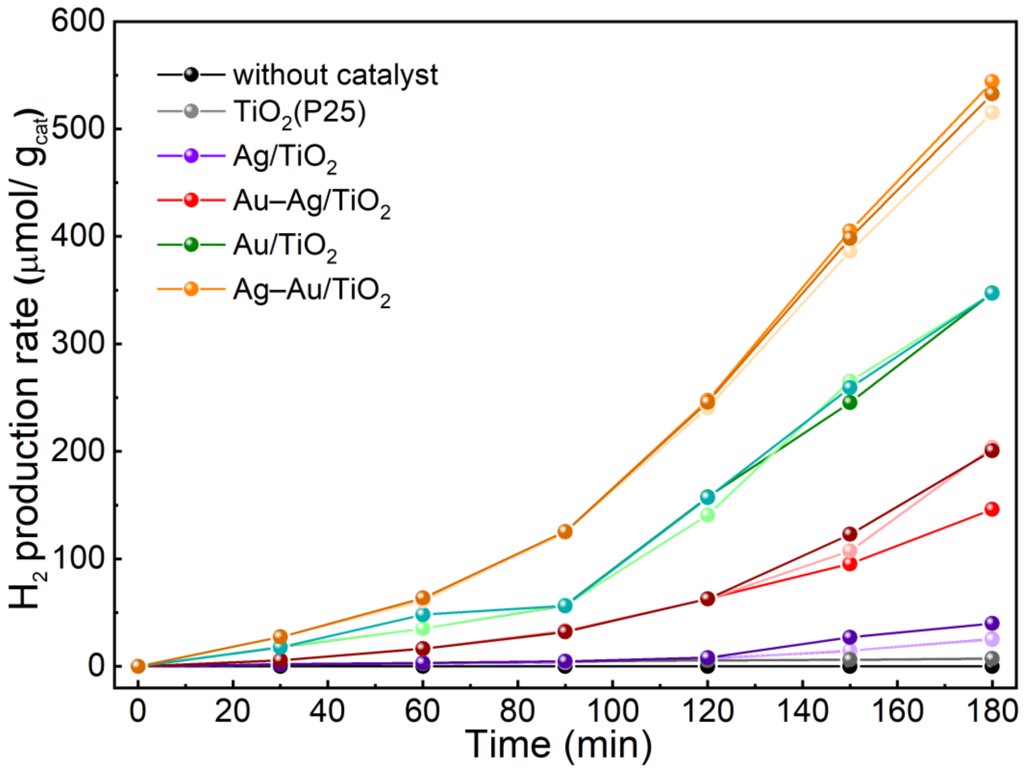

Figure S4. Reproducibility of photocatalytic hydrogen production reaction.

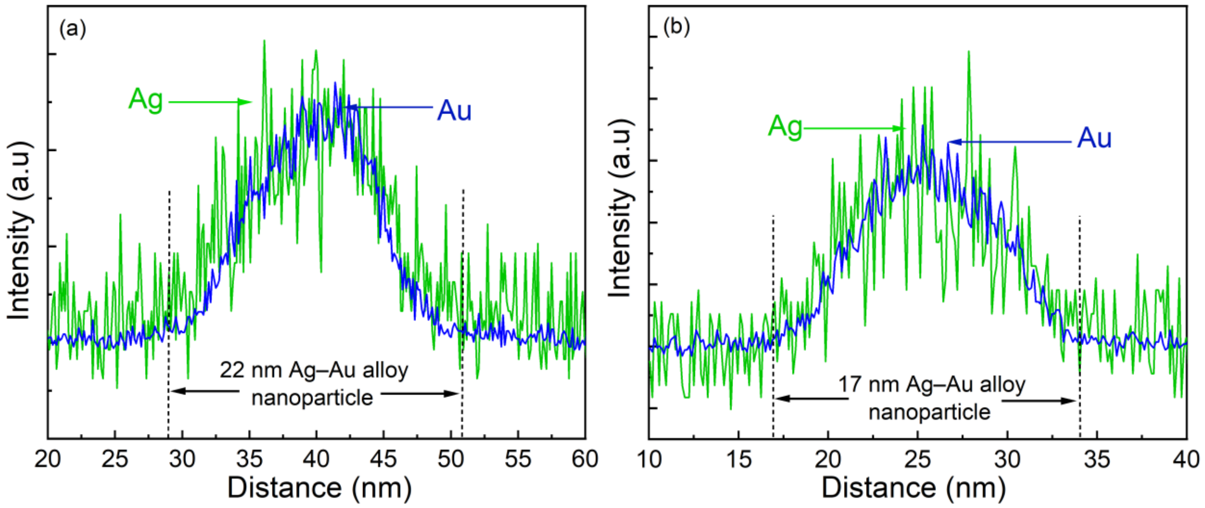

Figure S5. STEM-EDS line analysis of Ag–Au alloy nanoparticles on Au−Ag/TiO_2, i.e., samples on which Ag was photodeposited first.

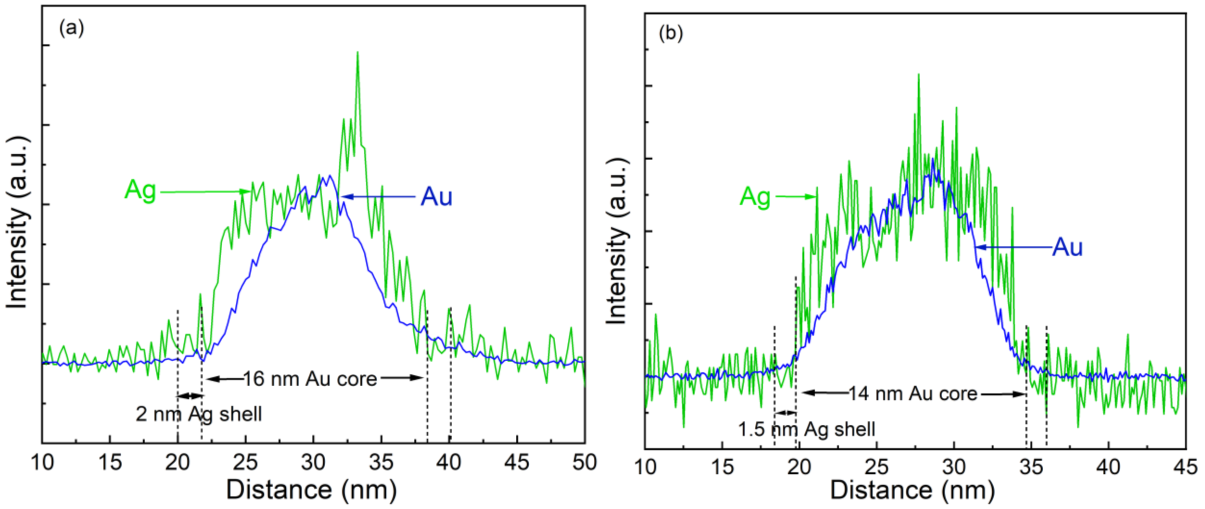

Figure S6. STEM-EDS line analysis of Ag-shell–Au-core nanoparticles on Ag−Au/TiO_2, i.e., samples on which Au was photodeposited first.
